# Public Attitudes Toward Anxiety Disorder on Sina Weibo: Content Analysis

**DOI:** 10.2196/45777

**Published:** 2023-04-04

**Authors:** Jianghong Zhu, Zepeng Li, Xiu Zhang, Zhenwen Zhang, Bin Hu

**Affiliations:** 1 Gansu Provincial Key Laboratory of Wearable Computing School of Information Science and Engineering Lanzhou University Lanzhou China

**Keywords:** anxiety disorder, linguistic feature, topic model, public attitude, social media

## Abstract

**Background:**

Anxiety disorder has become a major clinical and public health problem, causing a significant economic burden worldwide. Public attitudes toward anxiety can impact the psychological state, help-seeking behavior, and social activities of people with anxiety disorder.

**Objective:**

The purpose of this study was to explore public attitudes toward anxiety disorders and the changing trends of these attitudes by analyzing the posts related to anxiety disorders on Sina Weibo, a Chinese social media platform that has about 582 million users, as well as the psycholinguistic and topical features in the text content of the posts.

**Methods:**

From April 2018 to March 2022, 325,807 Sina Weibo posts with the keyword “anxiety disorder” were collected and analyzed. First, we analyzed the changing trends in the number and total length of posts every month. Second, a Chinese Linguistic Psychological Text Analysis System (TextMind) was used to analyze the changing trends in the language features of the posts, in which 20 linguistic features were selected and presented. Third, a topic model (biterm topic model) was used for semantic content analysis to identify specific themes in Weibo users’ attitudes toward anxiety.

**Results:**

The changing trends in the number and the total length of posts indicated that anxiety-related posts significantly increased from April 2018 to March 2022 (*R*^2^=0.6512; *P*<.001 to *R*^2^=0.8133; *P*<.001, respectively) and were greatly impacted by the beginning of a new semester (spring/fall). The analysis of linguistic features showed that the frequency of the cognitive process (*R*^2^=0.1782; *P*=.003), perceptual process (*R*^2^=0.1435; *P*=.008), biological process (*R*^2^=0.3225; *P*<.001), and assent words (*R*^2^=0.4412; *P*<.001) increased significantly over time, while the frequency of the social process words (*R*^2^=0.2889; *P*<.001) decreased significantly, and public anxiety was greatly impacted by the COVID-19 pandemic. Feature correlation analysis showed that the frequencies of words related to work and family are almost negatively correlated with those of other psychological words. Semantic content analysis identified 5 common topical areas: discrimination and stigma, symptoms and physical health, treatment and support, work and social, and family and life. Our results showed that the occurrence probability of the topical area “discrimination and stigma” reached the highest value and averagely accounted for 26.66% in the 4-year period. The occurrence probability of the topical area “family and life” (*R*^2^=0.1888; *P*=.09) decreased over time, while that of the other 4 topical areas increased.

**Conclusions:**

The findings of our study indicate that public discrimination and stigma against anxiety disorder remain high, particularly in the aspects of self-denial and negative emotions. People with anxiety disorders should receive more social support to reduce the impact of discrimination and stigma.

## Introduction

Anxiety disorder is a common mental disorder. Anxiety disorders involve repeated episodes of intense anxiety and sudden feelings of fear or terror, which peak in a few minutes (panic attacks). These feelings of anxiety and panic can impact the patient’s normal schooling, work, and life. Anxiety disorders are the most prevalent among adolescents, and according to the statistics of the World Health Organization, 3.6% of the adolescents aged 10-14 years and 4.6% of the adolescents aged 15-19 years worldwide experience anxiety disorders [1]. According to a report on the status of nutrition and chronic diseases in China, the prevalence of anxiety disorders in China reached 4.98% in 2020 [2]. In particular, with the spread of COVID-19, there is a substantial increase in the global cases of anxiety disorders. According to Santomauro et al [3], in a study of 204 countries and regions worldwide, the prevalence of global major depression increased by 26% in 2020.

Compared to people without anxiety disorders, those with anxiety disorders may have unstable interpersonal relationships, poorer functions, and higher rates of work absenteeism, with significant economic costs and impacts on physical health [4-6]. Moreover, anxiety disorders are associated with a significantly increased mortality risk. Compared with the general population, people with anxiety disorders have 1.4 times increased risk of death from natural causes and 2.5 times increased risk of death from nonnatural causes [7]. The high prevalence, chronicity, and the associated excessive mortality led the World Health Organization to rank anxiety disorders as the ninth leading health-related cause of disability [8], causing a significant economic burden, accounting for 3% of the global burden of disease worldwide and costing about €74.4 billion in 30 European countries [9].

Several factors prevent people from seeking help for mental illness, including poor quality of services, low levels of health literacy in mental health, and stigma and discrimination. Formal mental health services are not available in many places. Even if they do, they are often unavailable or unaffordable [10]. People often choose to endure mental suffering without relief, rather than risk discrimination and exclusion to access mental health services. The proportion of people with anxiety disorders who received any form of treatment is estimated to be 27.6% worldwide, but only 9.8% received adequate treatment [11]. Shame and stigma are the major obstacles for patients with anxiety disorders to receive an early diagnosis and professional treatment [12-14]. Patients with depression or anxiety disorders (compared to those without psychiatric disorders) are twice as likely to have a stigma, and the association between depression and anxiety is even stronger [15]. In addition, negative public attitudes may adversely impact patients’ social functioning (eg, interpersonal relationships, learning, work abilities) [16].

Public attitudes toward anxiety disorder can impact the psychology and daily lives of patients with anxiety. Most current research on attitudes toward mental illness use the method of questionnaire surveys [14,15,17]. This is because a questionnaire is an effective way of collecting information in research; other potentially richer measures of attitude, such as qualitative analysis, are often not feasible in large-scale studies due to labor costs [18]. However, the information collected by existing research methods is highly influenced by the subjective will of the investigators. In addition, the existing research methods are still limited in their ability to explore the structure of language expression, which would reflect people’s internal activities. To obtain information on the linguistic expressions of large groups of people, we turn our attention to the information available on social media. As an important medium of mass communication, social media contain a large amount of information that reflects people’s inner activities and emotional states. Users may often present their mental problems or illnesses anonymously through a wide variety of social media or online social health communities [19]. Such an online health community can be a network to express compassion by communicating with people who have similar symptoms [20]. In addition, users often try to obtain health information related to their symptoms on social media in an attempt to diagnose themselves [21,22]. Therefore, the posts on social media provide natural language data on people’s attitudes toward anxiety disorders.

A large number of studies in recent years have shown that social media data can be used to better understand, identify, and describe mental disorders [23,24] (eg, data from Facebook, Twitter, Instagram, Sina Weibo platforms). Individuals with mental disorders show changes in language and behavior, such as greater negative emotions and heightened self-attentive focus [25-28]. There is a high degree of similarity between patients with different forms of mental distress. Moreover, social media data can be used to assess the anxiety level of users [29,30], identify anxiety disorders [19,31], and assess the anxiety level of the public [32,33], thus enabling the assessment of the anxiety level of a large population of individuals or groups. These assessment methods might make up for the shortcomings of the traditional approach using questionnaires such as the Self-rating Anxiety Scale because it is difficult to ask a large number of individuals to fill in those questionnaires to estimate their anxiety levels separately. In addition, some studies showed that citizens’ perceptions and attitudes toward events can be tracked and discovered by analyzing the content posted on social media platforms [34,35]. Health-related stigmatizing attitudes (eg, depression, suicide) can be identified by analyzing the linguistic features expressed in social media posts [36-39]. Differences in stigmatizing attitudes toward mental health issues can also be reflected in different patterns of language use [40].

Social media–based research on attitudes toward mental illness is conducted at 2 main levels. The first level of analysis is at the text feature level, using word frequency statistics and affective tendency analysis to infer attitudes. Li et al [37] accurately discovered depression discrimination on social media through linguistic analysis methods. Li et al [40] constructed a model to distinguish schizophrenia-related stigma from depression-related stigma by using some psycholinguistic features automatically extracted from each post. Shen and Rudzicz [19] separated posts on 4 different anxiety-related subreddits from posts on control subreddits with an accuracy of 98%. They used a combination of N-gram language modeling and Linguistic Inquiry and Word Count (LIWC) [41].

The second level of analysis is topic-based analysis. Compared to text feature analysis, topic-based analysis is a higher and a more meaningful level of research. Topic models are divided into qualitative topic modeling methods and quantitative topic modeling methods. The qualitative topic analysis includes mainly content analysis methods (eg, NVivo software, QSR International), which is a systematic method to summarize the expressed content by inference from the text [42]. Reavley and Pilkington [43] used content analysis methods to find that tweets related to depression and schizophrenia were mostly supportive or neutral, and more than one-third of the tweets reflecting stigmatized attitudes toward depression were mocking or belittling patients. Lachmar et al [44] used the qualitative content analysis method to code tweets containing the tag #My depression looks like# on Twitter in May 2016. The content analysis revealed 7 themes: dysfunctional thoughts, lifestyle challenges, social struggles, hiding behind a mask, apathy and sadness, suicidal thoughts and behaviors, and seeking relief. Although the qualitative analysis method is effective, it usually requires researchers to have clinical expertise, which limits the wide use of the method.

The main methods of quantitative topic analysis include Latent Dirichlet Allocation (LDA) and its improvement methods. LDA is a statistical model that uses a data-driven unsupervised machine learning process to discover the underlying semantic structure from a series of documents. The latent semantic structure consists of a set of related topics that are identified by the words of co-occurrence. The results of the analysis are presented as word sets of co-occurrence, whose common themes are inferred by the researcher [45,46]. Franz et al [47] used the LDA model to detect content in social media containing self-harming thoughts and self-harming behaviors. Liu and Shi [28] used the LDA model to find that there were 7 main topics discussed by depressed patients on the Sina Weibo platform: negative emotion fluctuation, disease treatment and somatic responses, sleep disorders, sense of worthlessness, suicidal extreme behavior, seeking emotional support, and interpersonal communication. Jo et al [48] used a Structural Topic Model similar to the LDA model [49] to analyze users’ anxiety and worry concerns by analyzing data from 13,148 questions and 29,040 answers related to COVID-19 on Naver, a social networking platform in South Korea, and by using a structured topic model and a method of analyzing the network of words. They found that the long-term situation resulted in a slight increase in the proportion of work-related topics and that people’s anxiety and worry are closely related to physical symptoms and methods of self-protection. Paul and Dredze [50] obtained 13 health-related topics in Twitter data by constructing the Ailment Topic Aspect Model. Sik et al [51] used both quantitative (LDA topic modeling) and qualitative (deep reading) methods to determine the optimal number of topics and their interpretation in depression forums.

Previous studies on mental disorder attitudes on social media have described attitudes statically at the level of textual features and topic features. There are fewer studies on the dynamics of attitudes over time. Yu et al [38] studied public attitudes toward depression and the changes in these attitudes over time by combining the textual feature level and the topic level. The analysis of linguistic features showed that the frequency of use of emotion, positive emotion, anger, cognition (including the insight subcategory), and conjunctions increases significantly over time. The topic feature results suggested an upward trend in social support for people with depression over time, although there is a persistent stigma attached to depression. Using data from the Reddit platform, Low et al [52] analyzed trends from 90 text-derived features and found that many features including categories such as economic stress, isolation, and home increased significantly during COVID-19, while other categories such as motion decreased significantly. They also combined the LDA model and clustering algorithms and found a significant increase in health anxiety topics in midpandemic posts compared to those in prepandemic posts, with a large number of posts related to health anxiety topics expressing concerns with daily life at home, school, and work. Table 1 summarizes the research objectives and analytical methods of the existing work. For more research on the application of text analysis and natural language processing methods in health care, see [53,54].

In this study, we explored public attitudes toward anxiety disorders on social media and the changes in the attitudes over time at 2 levels: text features and topic features. Sina Weibo is the most popular social media platform in China, known as the Chinese version of Twitter. With 582 million monthly active users in 2022, Sina Weibo is a valuable source of information for studying attitudes toward anxiety. The purpose of this study was to use this rich data source to study public attitudes toward anxiety and how these attitudes change over time by analyzing the textual features and topic structure of publicly available posts on Chinese social media.

**Table 1:** A summary of the research objectives and analytical methods described in studies to date.

Study	Objective(s)	Analytical methods
Li et al [37]	Discover depression discrimination on social media	Text feature analysis
Li et al [40]	Distinguish schizophrenia-related stigma from depression-related stigma	Text feature analysis
Reavley and Pilkington [43]	Analyze the emotional polarity in tweets related to depression and schizophrenia	Qualitative content analysis
Lachmar et al [44]	Reveal the themes on tweets containing the tag #My depression looks like# in May 2016	Qualitative content analysis
Franz et al [47]	Detect content in social media containing self-harming thoughts and self-harming behaviors	Latent Dirichlet Allocation model (quantitative topic analysis)
Liu and Shi [28]	Reveal the topics talked about by depressed patients on the Sina Weibo platform	Latent Dirichlet Allocation model
Jo et al [48]	Analyze users’ anxiety and worry concerns by analyzing data related to COVID-19 on Naver platform	Structural Topic Model and the network of words
Paul and Dredze [50]	Reveal the health-related topics in Twitter	Ailment Topic Aspect Model
Sik et al [51]	Determine the optimal number of topics and their interpretation in depression forums	Latent Dirichlet Allocation model and qualitative analysis
Low et al [52]	Analyze health anxiety topics in midpandemic posts compared to those in prepandemic posts	Text feature analysis, Latent Dirichlet Allocation model and clustering algorithm
Yu et al [38]	Analyze public attitudes toward depression and the changes in these attitudes over time	Text feature analysis and Latent Dirichlet Allocation model

## Methods

### Data Collection

Sina Weibo is one of the most influential Chinese social media platforms. We use the application programming interface of Sina Weibo to obtain posts containing the keyword “焦虑症 (anxiety disorder),” which were posted from April 1, 2018, to March 31, 2022. We collected 403,132 Weibo posts. By a statistical method, we found that these posts were from 295,905 accounts, which means that about 5 in every 10,000 Sina Weibo users were engaged in conversations about anxiety disorders. To better reveal the textual features and topic structure of Weibo posts, before performing the analysis, we preprocessed the original posts according to the following steps:

1. We deleted the information posted automatically by organizations or Sina Weibo platforms.
2. We deleted users’ names mentioned by the symbol “@” in the main body of the posts.
3. We deleted English letters and emoticons.
4. The keywords “topic” and “super topic” of the posts contain the symbol “#” before and after the body, for example, “#super topic of anxiety#;” so, we used regular expressions to remove all the titles of topics and super topics that appeared in the posts.
5. We deleted advertising posts whose content recurred many times.
6. Finally, we deleted posts with very limited information, such as those containing only keywords.

Through the above preprocessing steps, we finally obtained 325,807 posts with the keyword “anxiety disorder.” To analyze the trend of the number of posts over time, we divided all the posts by month.

### Ethical Considerations

All the data in this paper were obtained from Sina Weibo’s public data, which protects those who have private profiles from being subject to research studies. Hence, this analysis meets the standards to waive informed consent and similar guidelines [55]. Furthermore, we desensitized the data to protect the privacy of the users. Specifically, we removed all individual information related to the identity of the users.

### Methods of Analysis

First, the preprocessed posts were divided by month to obtain time series data, and then we analyzed the changes in the number of posts related to anxiety disorders by using a linear regression method (R language; R Core Team and the R Foundation for Statistical Computing). Time series analysis can provide a reasonable mathematical model for the sample data to analyze the information and patterns contained in the sample time series. A time series usually has the following characteristics:

1. The positions of the data in the data series are determined by the temporal order, and there is a temporal correlation between them.
2. The values of different points in the data series are somewhat random; therefore, it is difficult to predict completely and accurately from historical observations.
3. The values with a relatively close time interval have a strong correlation, and this correlation can reflect the system evolution pattern.
4. Time series data usually tend to have some sort of trend and periodicity, such as seasonality.

We grouped the text data of the posts by month, from April 1, 2018, to March 31, 2022, and we fit a linear regression by using the number of posts and the total length of posts per month as a function of time. In addition, by analyzing these posts, we found that the number of posts containing the keyword “anxiety disorder” increased dramatically at the beginning of each new term. Approximately 10.67% (34,771/325,807) of the posts contained the keyword “new term begins.” To more clearly analyze the changing trend of the information contained in the posts with the keyword “anxiety disorder,” we selected a subset of the posts that contained no keyword “开学 (new term begins),” which included 291,036 posts (Table S1 in Multimedia Appendix 1).

Second, we performed textual analysis on the linguistic features of all the posts. The Chinese psychoanalysis tool TextMind [56] was used to conduct statistical analysis on high-frequency words related to the psychological characteristics in posts. TextMind can automatically segment words, classify words, and calculate the frequency of each category of word, using the Chinese lexicon named C-LIWC [57]. C-LIWC is a lexicon containing 32 linguistic features, 32 psychological features (belonging to 6 major categories), and 38 other features such as punctuation marks. In this study, 5 major categories were selected to analyze the trends and relevance characteristics of each category, and further, 11 subcategories of these 5 categories and 4 additional categories were selected to analyze the fine-grained changes (See Table 2 for details).

**Table 2:** Categories of the mental characteristics selected in the Linguistic Inquiry and Word Count dictionary.

Category	Abbreviation	Examples
Social process	Social	invite, hear, instruct, community, interact, public, culture
Family	Family	son, daughter, husband, parents, uncle, cousin, family
Affective process	Affect	serious, excessive, willing, rich, hope, promise, cope
Positive emotion	PosEmo	affectionate, loving, welcome, praise, glorious, interesting, kind
Negative emotion	NegEmo	resentful, heartless, failure, worry, trash, protest, abuse
Anxiety	Anx	restless, impatient, insomnia, fright, panic, anxiety, nervous
Anger	Anger	resentful, angry, enemy, fight, criticize, rage, agitated
Sadness	Sad	pitiful, disappointed, inferior, sorrowful, suffering, helpless, sad
Biological process	Bio	sick, fever, healing, tired, pain, numbness, vessels
Body	Body	finger, skin, ear, perception, breath, eye, shirt
Health	Health	live, care, insomnia, wound, surgery, health, scar
Cognitive process	CogMech	according, evidence, generally, intend, notice, otherwise, however
Insight	Insight	exactly, seem, think, admit, notice, believe, feeling
Exclusive	Excl	regardless, rather, if, otherwise, unless, suppose, however
Perceptual process	Percept	say, show, watch, listen, feel, touch
Feel	Feel	feel, soft, comfort, fuzzy, sharp, smooth, touch
(Others) I	I	I
(Others) Assent	Assent	hah-hah, alright, okay, yes, indeed, sure, clear, good
(Others) Work	Work	work, research, postgraduate, study, colleague, interview, unit
(Others) Achievement	Achieve	success, accomplish, achieve, encourage, reward, plan, effect

The Chinese psychological analysis system TextMind is a software system developed by the Computational Network Psychology Laboratory of the Institute of Psychology, Chinese Academy of Sciences, for linguistic analysis of Chinese text. TextMind provides users with a full set of analysis solutions from automatic word segmentation in simplified Chinese to linguistic psychological analysis; its lexicon, text, and symbol processing methods are specifically tailored to the simplified Chinese context, and its lexicon classification system is also compatible with LIWC.

Finally, we used a topic model to analyze the topics of all text data on Sina Weibo for each month. The topic model can generate keywords in each topic and the occurrence probability of each topic appearing in the entire document according to the predetermined number of topics. The LDA and the biterm topic model (BTM) are the classic topic models. The BTM [58] uses biterm (word pairs) to model the text, which can better display the hidden topics in the article; so, the BTM is more suitable for short-text modeling and analysis. Therefore, we chose the BTM to analyze the topics discussed in anxiety-related posts. In the preliminary analysis, the number of topics in BTM was set to 30.

## Results

### Trend Analysis of the Number and Total Length of Sina Weibo Posts

**Figure 1:**
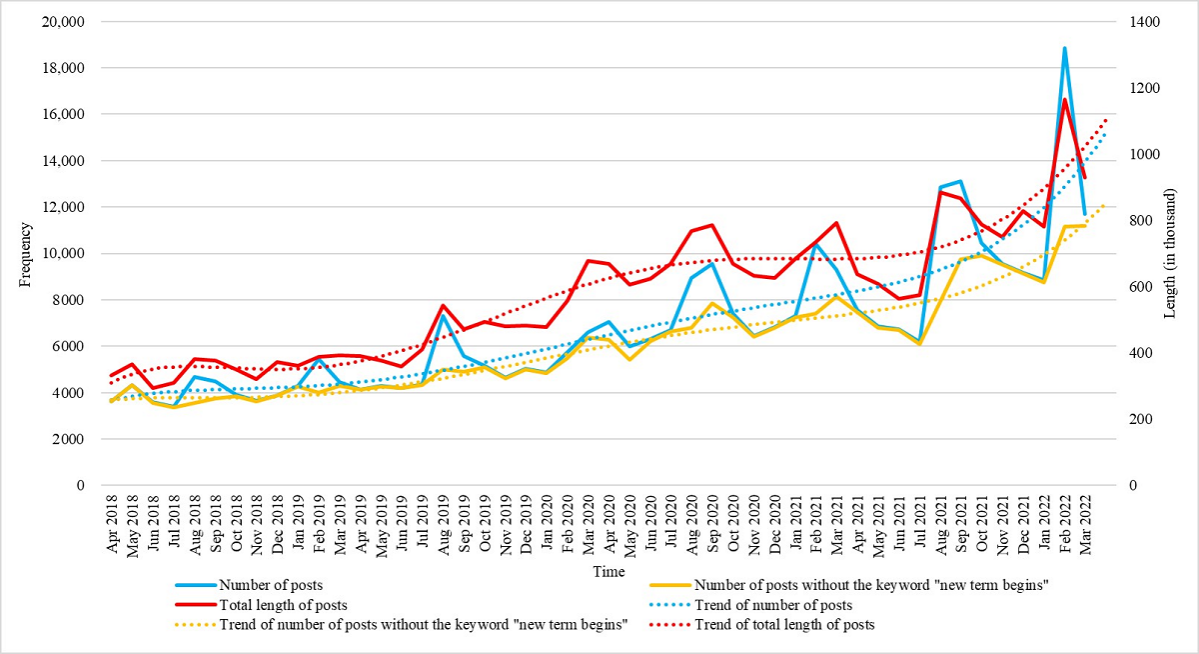
The trend in the number and total length of Sina Weibo posts.

Figure 1 shows the changes in the number and total length of anxiety-related posts from April 2018 to March 2022, as well as the changes in the number of posts excluding the keyword “new term begins.” The number of all anxiety-related posts fluctuated over time and showed a clear upward trend, especially after the outbreak of COVID-19. The upward trend was more clearly illustrated by the changes in the number of posts excluding the keyword “new term begins.” The number of posts increased significantly each year before the start of new terms. However, due to the epidemic, the start of the spring semester in 2020 was postponed to March or April; so, there was no obvious peak. The trend in the total length of anxiety-related posts was almost identical to the trend in the number of posts. Moreover, the increase in the total length of posts was even more remarkable in the early stages of the pandemic. Table 3 shows the results of linear regression. The estimate index in Table 3 shows that regardless of whether there were all anxiety-related posts or anxiety-related posts without the keyword “new term begins,” the changes over time showed a significant increasing trend. The *R*^2^ value of the anxiety-related posts without the keyword “new term begins” was 0.8671, indicating that the linear regression model had a better fit.

**Figure 2:**
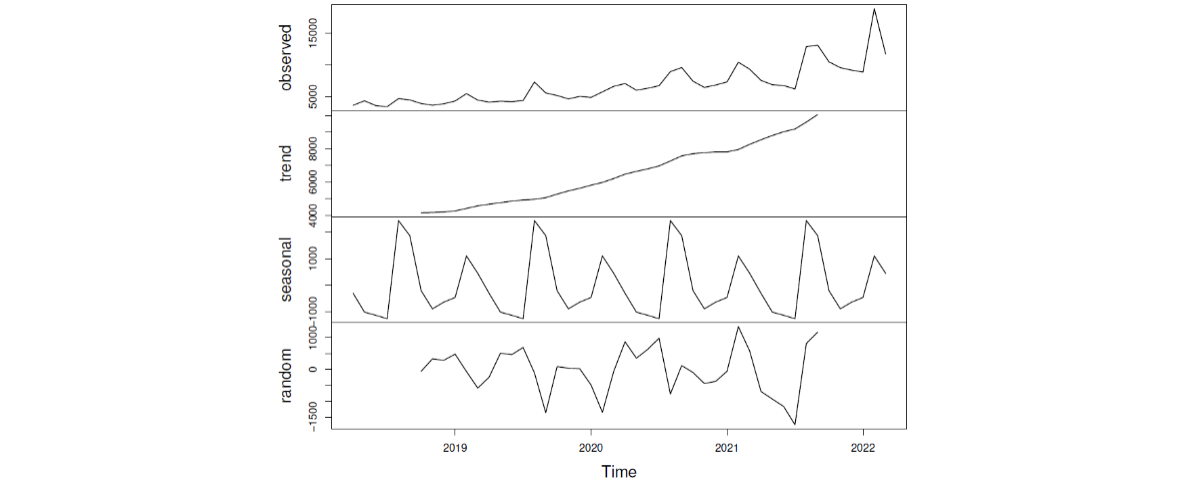
Time series decomposition of the number of Sina Weibo posts.

In addition, we used the time series decomposition function [59] to obtain the long-term trend, seasonal trend, and random term of the number of anxiety-related posts (see Figure 2). Similar to the results in Table 3, the long-term trend presented an obvious upward trend. In the seasonal trend, there were 2 peaks every year in February and August, but the peak in August was higher. Besides, in China, February and August happen to be the beginning of a new semester (spring/fall) every year, and August is also the beginning of the new school year. Thus, we can conclude that the beginning of new semester has a significant impact on anxiety in Sina Weibo posts.

**Table 3:** Linear regression results of the number of Sina Weibo posts.

Results	Predictor	Estimate	SE	*t* test *(df)*	*R* ^2^	*P* value
Number of posts	Time	176.83	19.08	9.267 (46)	0.6512	<.001
Number of posts without the keyword “new term begins”	Time	141.34	8.158	17.32 (46)	0.8671	<.001
Total length of posts	Time	1.2762	0.090	14.16 (46)	0.8133	<.001

### Analysis of Linguistic Features

#### Analysis of the Features of Major Categories of Words

**Figure 3:**
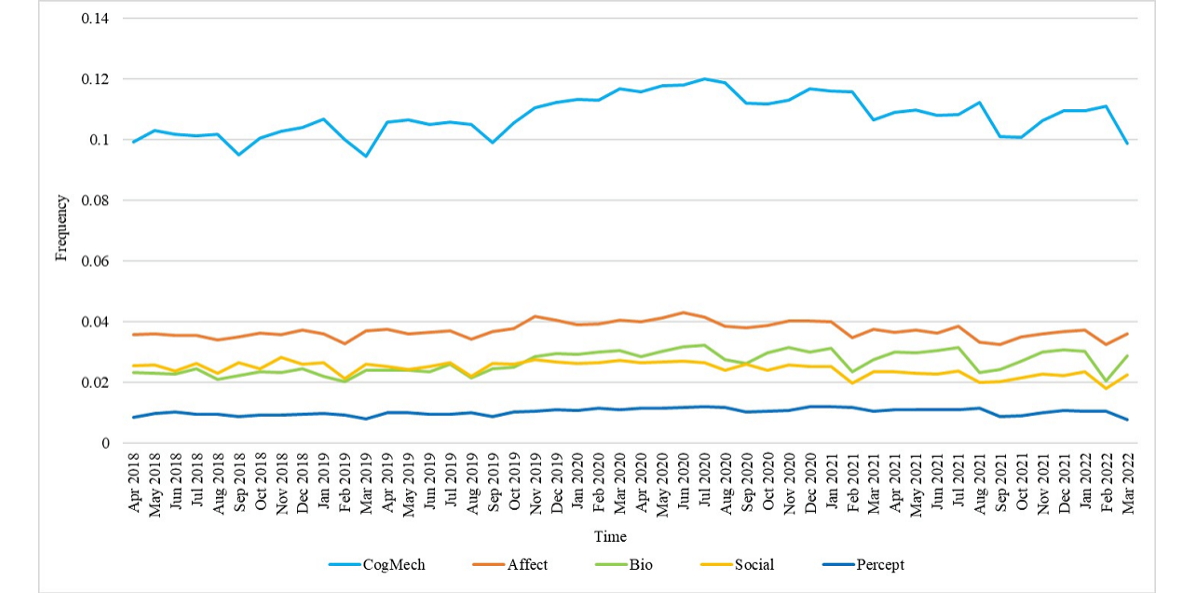
Occurrence frequency of the 5 major linguistic feature words: Social, Affect, Bio, CogMech, and Percept. Affect: affective process; Bio: biological process; CogMech: cognitive process; Percept: perceptual process; Social: social process.

As shown in Table 2, 5 major categories of psychological features were chosen: social process (Social), affective process (Affect), biological process (Bio), cognitive process (CogMech), and perceptual process (Percept). We analyzed the occurrence frequency of these 5 categories of words in anxiety-related posts from April 2018 to March 2022 (Table S2 in Multimedia Appendix 1); the results are shown in Figure 3. The words related to CogMech appeared most frequently, followed by those related to Affect, Bio, and Social. The frequency of words related to Percept was the lowest, and the change was not obvious during the 4-year period. The frequency of the words related to CogMech, Affect, and Bio increased during the early stage of COVID-19, from January 2020 to July 2020, while the frequency of the words related to Social decreased. Furthermore, Table 4 shows the linear regression results of the frequency of these 5 major categories of words. The words related to Social showed a downward trend, while the other 4 categories of words showed an upward trend, and the use of words related to CogMech and Bio had the most obvious increasing trend. Moreover, the frequency of these 5 categories of words fluctuated greatly over time; therefore, the *R*^2^ values were all small under the linear regression fitting model.

**Table 4:** Linear regression results of the occurrence frequency of the 5 major linguistic feature words.

	Predictor	Estimate	SE	*t* test *(df)*	*R* ^2^	*P* value
Social^a^	Time	–8.907e-05	2.060e-05	–4.323 (46)	0.2889	.001
Affect^b^	Time	1.224e-05	2.622e-05	0.467 (46)	0.0047	.64
Bio^c^	Time	1.452e-04	3.102e-05	4.680 (46)	0.3225	<.001
CogMech^d^	Time	1.967e-04	6.229e-05	3.158 (46)	0.1782	.003
Percept^e^	Time	2.942e-05	1.060e-05	2.776 (46)	0.1435	.008

^a^Social: social process.

^b^Affect: affective process.

^c^Bio: biological process.

^d^CogMech: cognitive process.

^e^Percept: perceptual process.

#### Analysis of the Features of Subcategories of Words

We selected 11 subcategories out of the 5 major categories (see Table 2) and the other 4 subcategories (I, assent, work, and achieve) to analyze the changing patterns of the various categories of words (Table S2 in Multimedia Appendix 1) and their correlation at a fine-grained level. Table 5 shows the linear regression results of the 15 subcategories of the features. Table 5 demonstrates that the use of words related to negative emotion (NegEmo), anxiety, anger, sadness, body, health, exclusive, feel, I, and assent was on the rise, while the use of words related to family, positive emotion (PosEmo), insight, work, and achievement was on the decline.

**Figure 4:**
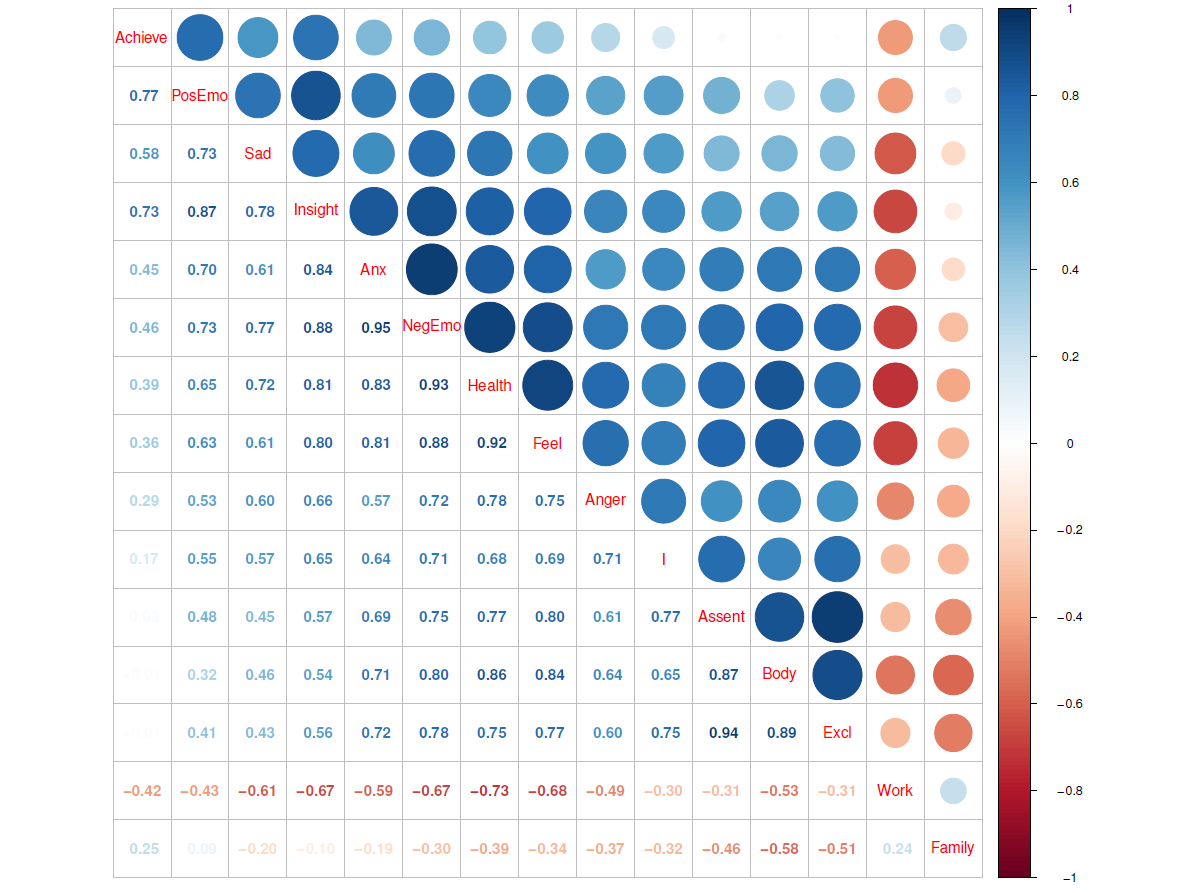
Correlation results among the 15 subcategories of features. Achieve: achievement; Anx: anxiety; Excl: exclusive; NegEmo: negative emotion; PosEmo: positive emotion; Sad: sadness.

Figure 4 shows the correlation results among the 15 subcategories of features. As shown in the figure, the words related to work and family were almost negatively correlated with other features. Anxiety had the highest correlation with NegEmo, followed by assent with exclusive, NegEmo with health, and health with feel. The features with the highest negative correlation were work and health. In addition to NegEmo, there were insight, health, and feel—all of which were highly correlated with anxiety.

**Table 5:** Linear regression results of the 15 subcategories of the 5 features.

	Predictor	Estimate	SE	*t* test *(df)*	*R* ^2^	*P* value
Family	Time	–2.252e-05	3.775e-06	–5.964 (46)	0.4361	<.001
Positive emotion	Time	–1.623e-05	1.088e-05	–1.491 (46)	0.0461	.14
Negative emotion	Time	3.776e-05	1.516e-05	2.491 (46)	0.1189	.02
Anxiety	Time	1.612e-05	8.281e-06	1.947 (46)	0.0761	.06
Anger	Time	3.611e-06	1.331e-06	2.712 (46)	0.1378	.009
Sadness	Time	7.044e-07	2.888e-06	0.244 (46)	0.0013	.81
Body	Time	8.533e-05	1.093e-05	7.809 (46)	0.5700	<.001
Health	Time	5.264e-05	1.685e-05	3.123 (46)	0.1750	.003
Insight	Time	–4.903e-07	1.204e-05	–0.041 (46)	3.61e-05	.97
Exclusive	Time	7.328e-05	1.068e-05	6.861 (46)	0.5058	<.001
Feel	Time	1.281e-05	4.384e-06	2.922 (46)	0.1566	.005
I	Time	4.865e-05	1.254e-05	3.880 (46)	0.2466	<.001
Assent	Time	7.493e-05	1.243e-05	6.027 (46)	0.4412	<.001
Work	Time	–1.938e-05	3.645e-05	–0.532 (46)	0.0061	.60
Achievement	Time	–3.314e-05	8.021e-06	–4.132 (46)	0.2707	<.001

### Topic Analysis of Sina Weibo Posts

We performed topic modeling on 325,807 anxiety-related posts from April 2018 to March 2022 by month. Since the setting of the number of topics in the topic model will impact the effect of the model, we compared the results of the models with 20, 30, 50, and 100 topics, and finally, we selected the model with 30 topics for further analysis. In addition, we selected a subset of topics that were easy to infer and mark their meanings. Then, we divided the topics into 5 areas: (1) discrimination and stigma, (2) symptoms and physical health, (3) treatment and support, (4) work and social, and (5) family and life (Table S3 in Multimedia Appendix 1). Each area included at least 3 related topics.

**Figure 5:**
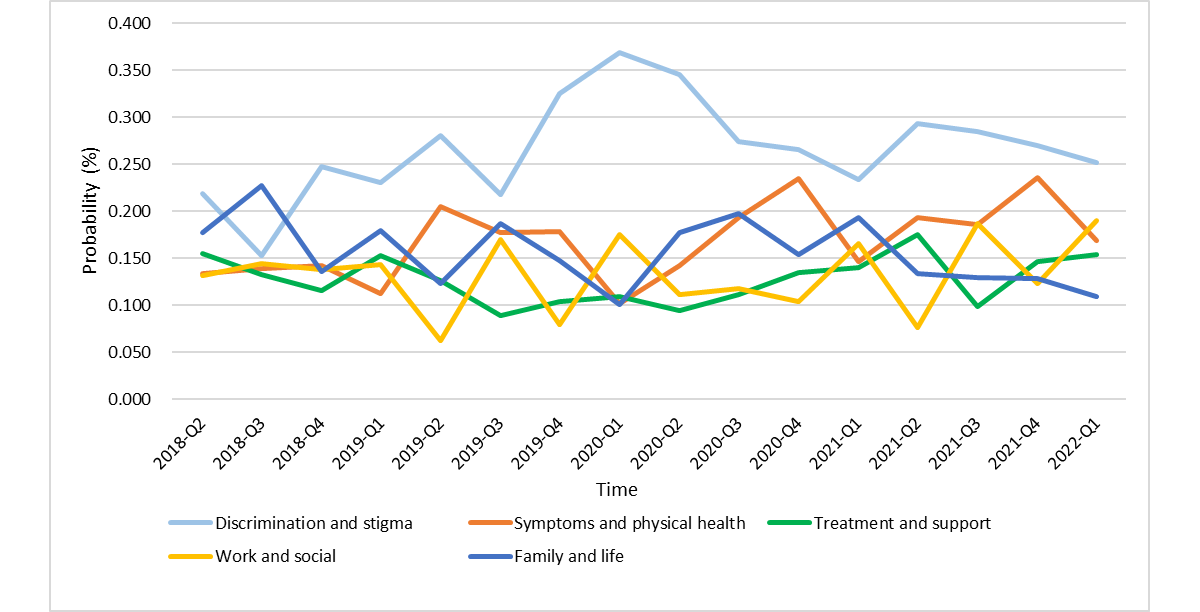
Average quarterly probability of occurrence of the 5 topics in 325,807 anxiety-related posts from April 2018 to March 2022.

To make the changing trend of occurrence probability in each area clearer, we calculated the probability of occurrence in each month in each area first and then calculated the average probability of occurrence in each quarter in each area (Figure 5). The results showed that from April 2018 to March 2022, the topical area with the highest probability of occurrence was discrimination and stigma, followed by symptoms and physical health, family and life, and work and social; the lowest was treatment and support. Especially, in the early stage of COVID-19, the occurrence probability of the topical area “discrimination and stigma” reached the highest value, while the occurrence probability of the topical areas “symptoms and physical health” and “family and life” both decreased, reaching almost the lowest value in 4 years. Specifically, the topical area related to discrimination and stigma averagely accounted for 26.66% in the 4-year period and 37.75% in the early stage of COVID-19.

Table 6 shows the linear regression results of the occurrence probability of the 5 topical areas. It can be seen that “symptoms and physical health” had a significantly positive trend, while “family and life” had a significantly negative trend. In general, combining the results of linguistic feature analysis and topic model, we observed the following results: first, the level of anxiety among Weibo users was significantly affected by the beginning of new terms, which may be due to the high proportion of teenage users. The topic model shows that “work and social” had obvious periodicity, with a high occurrence probability in the first and third quarters of each year and a low occurrence probability in the second and fourth quarters. According to the trend analysis of the number and total length of posts, this area was greatly impacted by topics related to “new term begins.” Second, the public was more anxious around the spring festival. Treatment and support had an obvious upward trend in 2019 and the first quarter of 2021, reaching the minimum in the third quarter. The upward trend was not obvious in early 2020 because of COVID-19, but it still had an upward trend. Third, in the early stage of COVID-19, the public paid more attention to discrimination and stigma, while “symptoms and physical health” and “family and life” showed a significant downward trend. This is consistent with the analysis of the linguistic features in Table 4 that the frequency of CogMech, Affect, and Bio increased, while the frequency of Social decreased. It is worth noting that the public can view anxiety in a positive light, with the topic model showing that family and life is inversely correlated with discrimination and stigma by the changing trend. From the results of the linguistic feature analysis, work and family were negatively correlated with almost all other features. This suggests that people should focus more on family and life to reduce discrimination and stigma.

**Table 6:** Linear regression results of the probability of occurrence of the topics.

	Predictor	Estimate	SE	*t* test *(df)*	*R* ^2^	*P* value
Discrimination and stigma	Time	4.00e-03	2.78e-03	1.440 (14)	0.1291	.17
Symptoms and physical health	Time	4.36e-03	1.90e-03	2.302 (14)	0.2746	.04
Treatment and support	Time	7.65e-04	1.42e-03	0.538 (14)	0.02026	.60
Work and social	Time	1.48e-03	2.18e-03	0.678 (14)	0.03176	.51
Family and life	Time	–3.29e-03	1.82e-03	–1.805 (14)	0.1888	.09

## Discussion

### Changes in Anxiety-Related Sina Weibo Content Over Time

This study analyzes the changes in anxiety-related post content over time from 3 aspects: post quantity, text characteristics, and topic structure. The results of our study indicated the following:

1. Between April 2018 and March 2022, there was a clear increase in public interest in anxiety disorders, particularly in terms of symptoms and treatment. According to the trend analysis of the number and total length of posts, whether it is the total number of anxiety-related posts or the number of anxiety-related posts without the keyword “new term begins,” the number of posts increased over time. Moreover, symptoms and physical health showed a significantly positive trend according to the linear regression results of topic frequency in 5 fields.
2. Public anxiety was greatly impacted by COVID-19, particularly in terms of work and social life. From the analysis of the frequency of anxiety-related posts for the 5 categories, namely, Social, Affect, Bio, CogMech, and Percept, words related to CogMech appeared most frequently, followed by words related to Affect, Bio, and Social. The frequency of words related to Percept was the lowest, and the changes were not obvious during the 4-year period. At the beginning of COVID-19, the linear regression results of the frequency of the 5 major categories of linguistic features showed a downward trend for Social-related words, while the remaining 4 categories showed an upward trend, with the most obvious increase in CogMech-related words. According to the linear regression results of the 15 subcategories, NegEmo, anxiety, anger, sadness, body, health, exclusive, feel, I, and assent showed an upward trend, while family, PosEmo, insight, work, and achievement showed a downward trend.
3. The public perception of discrimination and stigma against anxiety disorders remained high, especially in terms of self-denial and negative emotions. According to the correlation results among the 15 subcategories in Figure 4, the correlation between anxiety and NegEmo was the highest. Moreover, the most frequent topic in the topic analysis was discrimination and stigma, which was the most discussed topic, especially in the early stage of COVID-19.

Notably, the topic model also showed that the proportion of posts referring to family and life was lower than that of posts referring to discrimination and stigma. However, according to the average occurrence probability of anxiety-related posts in the topic model, the 2 topics were inversely correlated by trend analysis. In addition, there was a clear seasonal pattern in the topic of symptoms and physical health. Figure 5 shows that the occurrence probability of the topical area “symptoms and health” increased from autumn to winter, and Figure 3 shows that there was a significant increase in the number of the words of Affect and Bio from autumn to winter. This may indicate that people’s anxiety increases from autumn to winter and that the public pays more attention to their health in the winter. This phenomenon was similar to the change in the depression degree among social media users [60]. Previous studies have shown that compared to the control group, users with mental disorders prefer to use first-person pronouns [61]. In this study, we found that the frequency of first-person pronouns in anxiety-related posts remained high and tended to increase over time. This phenomenon suggests that anxiety-related users pay more attention to their CogMech. In addition, our study found a high frequency and a significant increase in the occurrence frequency of the words related to assent in anxiety-related posts. Assent words are usually positively correlated with users’ positive emotions [62,63]. However, in social media, some assent words indicate users’ disdain or helplessness to some extent.

### Limitations

This study has 3 main limitations. First, social media users cannot represent the entire Chinese population. According to the 50th statistical report on China’s internet development [64], the number of internet users in China reached 1.051 billion by June 2022. Most of these users were adults aged 20-39 years. Concurrently, there were 362 million non–internet users, most of whom lived in rural areas without access to the internet (41.2% of non–internet users live in rural areas). Lack of internet usage skills, literacy restrictions, age factors, and inadequate equipment are the main reasons for non–internet users not to access the internet [64]. Therefore, our results cannot be generalized to non–social media users. The trends of attitude changing over time found in this study may be different from the real trend. Second, the data analyzed in this paper are only for posts containing the keyword anxiety disorder; therefore, some posts that do not contain the keyword but express attitudes toward anxiety disorders are ignored. Third, the results obtained by this simple filtering method are only the overall attitude of Sina Weibo users toward anxiety disorders, lacking fine-grained analysis and without considering the impact of changes in the number of Weibo users. Additionally, there are other social media platforms in China besides Sina Weibo, and the generalization of our findings should be considered cautiously.

### Conclusions and Recommendations

In this study, the attitudes of Weibo users toward anxiety disorder and the changes in attitudes over time were explored by analyzing the linguistic features and topical structure of anxiety-related posts on Sina Weibo from April 2018 to March 2022. The results of this study showed that public discrimination and stigma against anxiety was high and that public anxiety was greatly impacted by the beginning of a new term and COVID-19. More specifically, the topics related to discrimination and stigma averagely accounted for 26.66% in the 4-year period and 37.75% in the early stage of COVID-19. Approximately 10.67% (34,771/325,807) of the posts contained the keyword “new term begins,” and the number increased dramatically at the beginning of each new term. Fortunately, public awareness of the symptoms and physical health aspects of anxiety disorders has increased significantly over time. Therefore, social media can be used to provide resources and assistance to patients with anxiety disorders and improve social media activities related to discrimination and stigma reduction.

In the future, we can try to consider English words, emoticons, and other characters in posts to more accurately reveal public attitudes toward anxiety disorder. In addition, by adding more keywords in the process of data collection, more posts on anxiety topics can be acquired and analyzed, which may expand the scope of research and generate more meaningful results.

## Appendix

Multimedia Appendix 1 Supplementary data.

## Abbreviations

Affect: affective process
Bio: biological process
BTM: biterm topic model
CogMech: cognitive process
LDA: Latent Dirichlet Allocation
LIWC: Linguistic Inquiry and Word Count
NegEmo: negative emotion
Percept: perceptual process
PosEmo: positive emotion
Social: social process
